# Alternative splicing induces sample-level variation in gene–gene correlations

**DOI:** 10.1186/s12864-024-11118-z

**Published:** 2024-12-11

**Authors:** Yihao Lu, Brandon L. Pierce, Pei Wang, Fan Yang, Lin S. Chen

**Affiliations:** 1 Department of Public Health Sciences, University of Chicago, 5841 South Maryland Ave, MC2000, Chicago, IL 60637 USA; 2 Department of Human Genetics, University of Chicago, 920 E 58Th St, Chicago, IL 60637 USA; 3 Department of Genetics and Genomic Sciences, Icahn School of Medicine at Mount Sinai, 770 Lexington Ave, New York, NY 10065 USA; 4 Department of Biostatistics and Informatics, Colorado School of Public Health, University of Colorado Denver, 13001 E. 17Th Place, Aurora, CO 80045 USA

**Keywords:** Alternative splicing, Total expression, Isoform, Gene–gene correlation, Sample-level variation

## Abstract

**Background:**

The vast majority of genes in the genome are multi-exonic, and are alternatively spliced during transcription, resulting in multiple isoforms for each gene. For some genes, different mRNA isoforms may have differential expression levels or be involved in different pathways. Bulk tissue RNA-seq, as a widely used technology for transcriptome quantification, measures the total expression (TE) levels of each gene across multiple isoforms in multiple cell types for each tissue sample. With recent developments in precise quantification of alternative splicing events for each gene, we propose to study the effects of alternative splicing variation on gene–gene correlation effects. We adopted a variance-component model for testing the TE–TE correlations of one gene with a co-expressed gene, accounting for the effects of splicing variation and splicing-by-TE interaction of one gene on the other.

**Results:**

We analyzed data from the Genotype-Tissue Expression (GTEx) project (V8). At the 5% FDR level, 38,146 pairs of genes out of ∼10 M examined pairs from GTEx lung tissue showed significant TE-splicing interaction effects, implying isoform-specific and/or sample-specific TE–TE correlations. Additional analysis across 13 GTEx brain tissues revealed strong tissue-specificity of TE-splicing interaction effects. Moreover, we showed that accounting for splicing variation across samples could improve the reproducibility of results and could reduce potential confounding effects in studying co-expressed gene pairs with bulk tissue data. Many of those gene pairs had correlation effects specific to only certain isoforms and would otherwise be undetected. By analyzing gene–gene co-expression variation within functional pathways accounting for splicing, we characterized the patterns of the “hub” genes with isoform-specific regulatory effects on multiple other genes.

**Conclusions:**

We showed that splicing variation of a gene may interact with TE of the gene and affect the TE of co-expressed genes, resulting in substantial tissue-specific inter-sample variability in gene–gene correlation effects. Accounting for TE-splicing interaction effects could reduce potential confounding effects and improve the robustness of estimation when estimating gene–gene correlations from bulk tissue expression data.

**Supplementary Information:**

The online version contains supplementary material available at 10.1186/s12864-024-11118-z.

## Introduction

In the past decade, bulk tissue RNA-seq technology was widely used to quantify transcriptome variation from multiple samples. Bulk RNA-seq measures the total expression (TE) levels for each gene in a sample, summing over multiple isoforms of each gene in a mixed cell population of the sample [1, 2]. Recent studies have examined cell-type-specific gene–gene regulation of transcriptional expression levels in the genome [3], and it was also reported that estimating and accounting for cell-type composition in bulk tissue samples greatly improved the precision in analyses of gene expression data [4]. Besides cell type, alternative splicing (AS) is another factor that contributes to inter-sample variation in TE levels of genes and gene–gene correlation/co-expression/regulation. However, although AS is one of the most widespread mechanisms involved in gene regulation, its role in transcriptional regulation is still not fully understood.

**Fig. 1:**
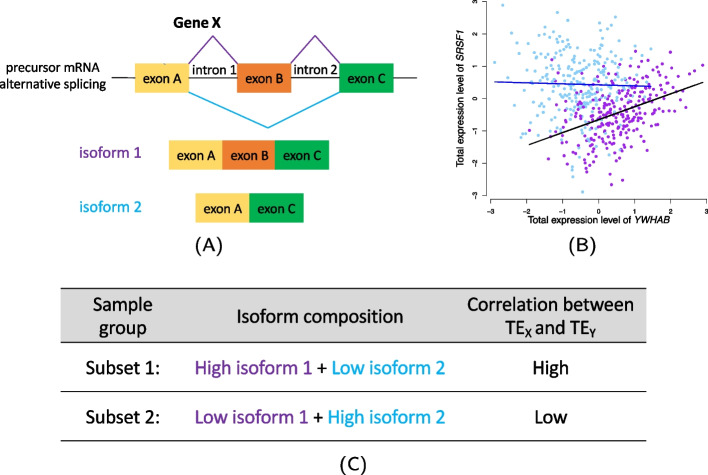
An illustration of how isoform-specific regulatory effects induce sample heterogeneity in TE–TE correlation. **A** Gene *X* was alternatively spliced and produced two isoforms. The two isoforms do not have differential transcriptional variation but have different regulatory effects on the transcription levels of gene *Y*, with isoform 1 having stronger effects. **B** For gene pair *SRSF1* and *YWHAB*, samples were stratified based on the proportion of one isoform of *YWHAB*. Blue samples had low proportion of the isoform while purple samples had high proportion. It could be seen from the figure that only samples with high proportion of the isoform had a non-zero TE–TE correlation (*P*-value < 0.001). **C** For samples that had similar TE levels of gene *X*, those that were enriched with isoform 1 (Subset 1) had much higher TE–TE correlations than samples with lower proportions of isoform 1 (Subset 2). Isoform-specific regulatory effects and sample-level variation in isoform composition induce inter-sample variation in TE–TE correlations

The vast majority of the genes in the human genome are multi-exonic (with a mean of 8*.*8 exons per gene [5]). Alternative RNA splicing is a critical step of gene regulation and allows a multi-exon gene to generate multiple isoforms with potentially different structures and functions. As illustrated in Fig. 1A, during transcription, certain exons of a multi-exonic gene may be included within or excluded from the final processed mRNA produced from the gene, resulting in different isoforms of the same gene. Splicing occurs frequently and ∼ 95% of multi-exonic genes are alternatively spliced [6]. Alternative splicing has greatly increased the biodiversity of human genome. A single gene may have different splicing events in different samples, resulting in different isoform composition for those samples. Differential isoform-level expression and regulation of genes/networks may also contribute to phenotypic variation including the development of complex diseases and particularly, cancers [7–9]. Splicing plays an essential role in cellular differentiation [10, 11]. In extreme cases, different combinations of multiple alternatively spliced regions can generate tens of thousands of isoforms from a single gene [12]. Different isoforms of the same gene may have distinct regulatory properties in the cell, have distinct downstream regulated genes with divergent functions, and be involved in different pathways and co-expression networks [13, 14]. In studying gene–gene correlation and regulation, it is important to account for expression and co-expression variation among samples due to splicing variation.

In studying gene–gene co-expression and regulation based on bulk tissue data, most existing analyses were based on estimating correlations and conditional correlations among TE levels of genes [15–17]. With the advancement of annotation-free quantification of RNA splicing variation, the quantification of isoform-level expression and splicing variation from raw sequencing reads of bulk tissue data was substantially improved [18]. In a recent study of multi-tissue expression data from the Genome-Tissue Expression project (v6p) [14], by combining TE levels and isoform ratios (IRs, i.e., relative isoform expression levels) of genes in the genome, a Transcriptome-Wide Network was built for each human tissue type to study the patterns of co-expression and regulation of splicing and total gene expression levels. The regulatory effects of TE-to-TE, TE-to-IR, IR-to-TE and IR-to-IR among genes in the genome for each tissue type were estimated based on a (modified) graphical lasso model [19]. The study provided a global view of the interplay among TEs and IRs, and examined “hub” genes and their degree centrality distributions across tissue types. Complimentary to existing global network construction, in this work we took a “bottom-up” approach and studied the interrelationships among TE and IR for pairs of genes in the genome and examined how splicing-induced isoform-specific effects may affect the analysis of co-expressed genes.

We analyzed the expression data from lung tissues (*N* = 515) of the GTEx (V8) project. We considered multiple isoforms of a gene as a set of variables for splicing variation. We observed that isoform composition of a gene often varied by sample, and the correlation effects of two co-expressed genes often depended on the isoform composition of the genes, showing inter-sample variability (or sample heterogeneity). In the illustrative examples shown in Fig. 1B, isoform 1 of the gene *YWHAB* had a non-zero effect on the expression level of the gene *SRSF1* and the effect is only specific to isoform 1. Since different samples have different proportion of isoform 1 of the gene *YWHAB*, samples with high proportions of isoform 1 of the gene had a much stronger TE–TE correlation with the gene *SRSF1*. In contrast, gene–gene correlations were much weaker in samples with low proportions of isoform 1 of *YWHAB*. The ubiquitous isoform-specific regulatory effects and the often sample-varying isoform composition induces inter-sample variation in the gene–gene correlation effects for different samples. Standard linear models assuming a same fixed gene–gene correlation for all samples may not adequately capture the co-expression/regulatory effects in bulk tissue data.

In this work, we adopted a mixed-effects model [20] in estimating the co-expression effects between pairs of genes while allowing sample-level variation as random effects, with the covariance matrix of the random effects being proportional to sample-sample covariance of isoform composition. The model can be generally applied to account for inter-sample variation in effects due to other sample-varying factors, including cell type composition and/or sets of covariates. The inter-sample variation in TE–TE correlation of two genes due to splicing variation in one of the gene (putative regulator gene) can also be considered as the interaction effect of splicing and TE of one gene on the co-expressed/regulated gene. We showed that accounting for splicing-induced sample-level variation could improve the power and precision in identifying gene–gene correlation. Moreover, we reconstructed co-expression networks accounting for splicing for known KEGG pathways [21] using data from lung tissue and brain tissues of the GTEx (V8) project and examined the distributions of degree centrality of “hub” genes in the networks. Splicing-induced variation and TE-splicing interaction effects tend to be tissue-specific. Gene pairs with significant TE-splicing interactions were found to have higher proportion of significant TE–TE correlations in both tumor and tumor-adjacent normal lung tissues from Clinical Proteomic Tumor Analysis Consortium (CPTAC). Furthermore, similar to cell-type aware analyses with bulk RNA-seq data, we showed that accounting for splicing variation would reduce potential unmeasured confounding effects when splicing affects both predictor and outcome in an analysis.

## Results

By analyzing expression data from lung tissue (*N* = 515) of the GTEx (V8) project [22], we found that inter-sample variation in TE–TE correlations (i.e., sample heterogeneity in gene–gene correlations) due to alternative splicing was prevalent in the genome. We showed that accounting for splicing variation could improve power and precision in detecting co-expressed gene pairs. We characterized the patterns of genes co-expressed with multiple other genes (i.e., “hub” genes) within specific KEGG pathways and their degree centrality distributions based on the significance of splicing-specific regulatory effects. Gene pairs found to have significant splicing-specific correlation effects in normal lung tissues from GTEx had a higher-than-random proportion of significant TE–TE correlations in lung tumor-adjacent normal tissues (*N* = 101) and tumor tissues (*N* = 110) of lung cancer patients from CPTAC, an independent dataset. Moreover, our results suggested that adjusting for splicing variation in analyzing TE with bulk tissue data would reduce potential confounding.

Studying inter-sample variation in TE–TE correlations due to alternative splicing with lung bulk tissue data from GTEx (V8).

There are 515 lung tissue samples in the GTEx (V8) project [22]. GTEx RNA sequencing was performed using the Illumina TruSeq RNA protocol. Data was aligned using STAR (v2.5.3a) [23]. Picard [24] was used to process raw sequence data. RNA-SeQC [25] was used for quality control and gene-level expression quantification, and TMM [26] was used to normalize read counts. Additional details on the RNA-Sequencing pipeline and processing are reported elsewhere [22]. We quantified the splicing events of each gene using LeafCutter [7, 18], an annotation-free quantification of RNA splicing method. Since alternative splicing involved different patterns of the removal of introns from pre-mRNA of a gene, Leafcutter quantifies the Intron Excision Ratios (IERs) as surrogate variables for isoform ratios of each gene. The number of IERs for each selected gene ranged from 2 to 46, with a median of 8 introns per gene.

We selected multi-exonic genes being expressed in lung tissues from both GTEx and CPTAC data. We further restricted the analysis to 3*,*223 genes included in at least one KEGG pathway [21]. There were a total of 3223 × 3222 = 10*,*384*,*506 gene pairs being considered in the pair-wise gene–gene correlation analysis.

We applied the mixed-effects model and the variance-component score test described in the Methods section to each pair of genes. The model considered the TE levels of one gene *Y* as the response variable, and the TE of a putative regulator gene *X* as the predictor gene. Additionally, the model included both a random slope and a random intercept for each sample with variance of random effects proportional to sample-sample IER covariance matrix, capturing inter-sample variation in the slope and intercept due to splicing variation among samples, respectively. Under the null, there was no random slope (all samples had the same fixed TE–TE correlation). In other words, splicing variation in *X* would not induce inter-sample variation in TE–TE correlation. Under the alternative, when at least one isoform of *X* had isoform-specific effect on the TE of *Y*, TE–TE correlation depended on the isoform composition of gene *X* in the samples (similar as shown in Fig. 1B). The variance-component score test described in Eq. 2 was used to detect inter-sample variation in effects due to splicing. In the analysis, we also adjusted covariates, including 5 genotype principal components (PCs), 60 PEER factors [27], sequencing platform, sequencing protocol and gender. Hereafter, we refer to the test in Eq. 2 for detecting inter-sample variation in effects due to splicing as the “interaction test” for TE-splicing interaction effects on the response gene *Y*.

**Fig. 2:**
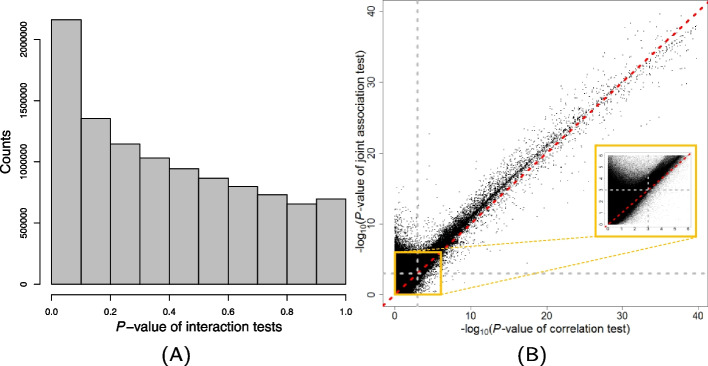
Analysis of 10, 384, 506 pairs of genes from 3, 223 genes from GTEx (V8) lung tissues (*N* = 515).** A** The histogram of *P*-values obtained from tests for inter-sample variation in TE–TE effects, i.e., TE-isoform interaction tests. **B** The scatter plot of − log_10_(*P*-values) from the standard correlation test (x-axis) versus − log_10_(*P*-values) from the joint association test (y-axis). Dashed lines corresponded to the *P*-value cutoff of 0.001 with suggestive evidences for significance. Points in the upper-left box showed the co-expressed gene pairs that were uniquely detected by the joint association test but not the standard correlation test. The inset shows a zoomed-in view of gene pairs close to the *P*-value cutoff

Figure 2A showed the histogram of *P*-values for the interaction test for the 10*,*384*,*506 tested gene pairs. The estimated non-null proportion [28] is 1 − *π*_0_ = 0*.*319, implying that a substantial proportion of gene pairs in the genome have heterogeneous correlation effects among different samples due to splicing variation. At a 5% FDR level, there were 38*,*146 pairs of genes found to be significant with inter-sample variation in TE–TE correlations. Even at the ultra stringent *P*-value cutoff of 4*.*8 × 10^−9^ by Bonferroni correction, there were still 153 significant gene pairs. Here we showed that the ubiquitous isoform-specific regulatory effects and sample heterogeneity in isoform composition induced inter-sample variability in gene–gene correlation. In contrast to existing literature restricting to the modulation of splicing variation in certain regulatory proteins on known transcription factors and their transcriptional target genes [29], our results showed that the modulation of splicing variation on the TE–TE correlation (interaction of splicing-TE on the TE of a target gene) is quite prevalent in the genome. Because many isoform-specific effects may not be strong and samples may not be enriched with certain isoforms with specific regulatory effects, the mixed-effect model and the variance-component score tests used in our analysis substantially improved the power for detecting the aggregated modulation effects due to splicing variation in genes [20, 30].

### Accounting for splicing variation improves the power for detecting gene–gene co-expression

In detecting gene–gene co-expression and regulation, it is a common practice to assess the correlations among TE levels of genes, which also serves as the backbone for constructing co-expression networks. Since alternative splicing may induce inter-sample variation in TE–TE correlations, we showed that by accounting for that variation and jointly testing for fixed and random effects of TE of one gene on the TE of another gene (both main effects and TE-splicing interaction effects of the predictor gene on the response gene), we observed power improvement in detecting co-expressed genes. When there is sample-level heterogeneity in effects (random slope), ignoring the random-effect may led to biased inference [31, 32]. In the Methods section, we described a variance-component score test for the joint effects of both fixed (main) and random (interaction) effects of the TE of one gene on the TE of another. We refer to the test in Eq. 3 for detecting both TE–TE correlation and TE-splicing interaction effects as the “joint association test”. We compared the power of the joint association test versus the power of the standard correlation test (main effects only) adjusting for the same set of covariates.

Applying a stratified 5% FDR threshold [33], a total of 1*,*200*,*242 pairs of co-expressed genes were significant based on the joint association test. In contrast, 93*,*304 pairs of gene were significant based on the standard correlation test (at the 5% FDR level). In Fig. 2B, we plotted the − log_10_(*P*-values) from the joint association test versus the − log_10_(*P*-values) from the standard correlation test. It can be seen that the two sets of *P*-values were highly correlated. Moreover, there are many gene pairs (left corner in Fig. 2B) which were significant in the joint association test only, implying strong splicing-specific TE–TE correlations among subsets of samples.

We further examined the relative contribution of variance in TE of gene *Y* explained by the fixed effects and random effects in the joint association test, measured by $\rho = \frac{{\widehat{\sigma }}_{\text{Int}}^{2}}{{\widehat{\sigma }}_{\text{Int }+ {\widehat{\sigma }}_{X}^{2}}^{2}}$ (see Eq. 5 in Methods). When *ρ* = 0, the joint association test is equivalent to the standard correlation test adjusting for covariates and IERs and there is no splicing-induced sample-level variation in TE–TE effect. When *ρ* is large, the splicing-induced random slope heavily affects the variation of TE of gene *Y*. For the 10*,*384*,*506 gene pairs being tested, the distribution of *ρ* showed a bi-modal pattern and was concentrated at zero with a median of 8*.*85 × 10^−6^. Among the 1*,*200*,*242 significant gene pairs based on the joint association test, the median of *ρ* was increased to 0*.*05. In other words, splicing-induced inter-sample variation was larger than random among co-expressed gene pairs. Furthermore, there were 37*,*572 gene pairs that were significant based on both the joint association test and the interaction test, and the median of *ρ* for these pairs was 0*.*76. Our results showed that the joint association test can identify both overall TE–TE correlations and TE–TE correlations specific to subsets of samples (with similar isoform compositions) and the estimated *ρ* is informative in characterizing the relative contributions of overall (fixed) TE–TE effects and isoform-specific TE–TE effects (interaction/random effects) when studying regulation patterns of co-expressed genes.

Analyses of co-expression pattern accounting for splicing-induced sample variation within specific KEGG pathways.

**Fig. 3:**
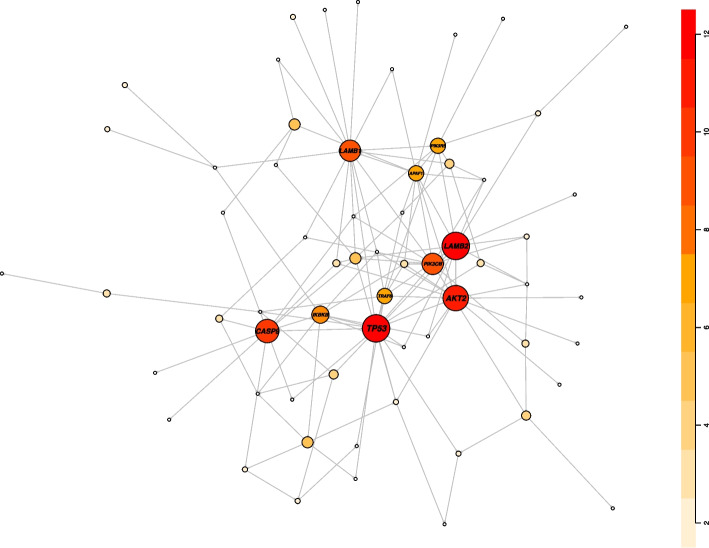
Network constructed based on the interaction test within the small cell lung cancer pathway from KEGG. The genes regulatory network was reconstructed based on the interaction test. Each directed edge in the figure represents significant TE-splicing interaction effects from one gene on the TE of another gene. *TP53* and *LAMB2* had the highest outdegrees, with TE-splicing interaction significantly affecting other 12 genes with the pathway. *AKT2* and *CASP9* also had significant TE-splicing interaction effects on more than 10 other genes

To examine the pattern of co-expression among functionally related genes accounting for splicing, we first analyzed the gene pairs from the small cell lung cancer pathway from KEGG [21]. In this pathway, there were 71 genes had at least 2 isoforms. We obtained the *P*-values for the 71 × 70 gene pairs based on results from the three tests: 1) the interaction test (testing for only splicing-induced sample-level variation in TE–TE correlations, i.e., TE-splicing interaction effects), 2) the joint association test (for both TE–TE correlation and TE-splicing interaction), and 3) the standard correlation test adjusting for covariates. Each network was constructed based on applying a 5% stratified FDR threshold [33] to all of the gene pairs. As shown in Fig. 3, based on the network from interaction tests, we detected two major hub genes: *TP53*, *LAMB2* with inter-sample variation in gene–gene correlation with many other genes in the pathway. The biggest hub *TP53* had significant TE-splicing interaction effects on the TE of 12 other genes. The gene *TP53* is well known for its role as a tumor suppressor [34].

Compared to the network constructed based on the standard correlation test (Additional file 1: Fig. S2A), the network based on the joint association test (Additional file 1: Fig. S2B) were more dense with more edges and hubs, though the hubs and structures were similar. Some hub genes, such as *BCL2L1*, *COL4A1*, *LAMC1* and *LAMB2* were shared by both networks. The network constructed by the joint association test was more complex and had bigger hubs that connected to up to 45 other genes. For shared hubs, the joint association network had generally higher outdegrees (here one outdegree was defined as the joint effect of TE and TE-splicing interaction being a significant predictor for the other gene). In contrast, the network based on the interaction test was relatively sparse. In Additional file 1: Fig. S3A, we further analyzed all 158 KEGG pathways and examined the degree centrality distributions of hub genes within each pathway, and reached similar conclusions. Considering that larger pathways had on average higher degrees, after adjusting for pathway size, in Additional file 1: Fig. S3B, we observed that the mean outdegrees per gene based on the joint association tests were higher than those based on the standard correlation tests, while the mean outdegrees per gene based on the interaction test was much smaller. The hub genes with splicing-induced effects tend to affect smaller communities. This could be at least partially attributed to power issues since interaction tests would be less powered than tests for main effects given the same sample sizes, while it was also possible that splicing-specific hubs tended to be less stable and as such less likely to form a major hub for many others.

### Gene pairs with splicing-specific TE–TE correlations have higher-than-random proportions of TE–TE correlations in both tumor and normal lung tissues from CPTAC

To examine whether splicing-specific TE–TE correlations can be replicated in other studies, we analyzed the TE–TE correlations in the lung adenocarcinomas tissues and the tumor-adjacent normal tissues from CPTAC [35]. The CPTAC program [36] systematically characterized the proteins and genes that derive from alterations in cancer genomes and related biological processes and provide this data to the public that allows a wider group of scientists to study the transcriptome and proteome variation in tumor tissues. Lung adenocarcinomas makes up about 40% of all lung cancers and is a leading cause of cancer-related mortality with more than a million deaths each year [35]. In this analysis, we separately analyzed the gene–gene TE–TE correlations in the lung adenocarcinomas tumor tissues (*N* = 110) and the tumor-adjacent normal tissues (*N* = 101) from CPTAC, adjusting for smoking status, cancer stage, western, age, male, height, weight, BMI, immune score, stromal score (only for normal tissues), mutation and fusion matrix, as well as surrogate variables calculated by the sva [37] package in R (10 surrogate variables for tumor tissues and tumor-adjacent normal tissues respectively). At the 5% FDR level, 157*,*726 pairs of genes were significant in tumor tissues and 181*,*600 pairs were significant in tumor-adjacent normal tissues. For the 3*,*002 gene pairs that had insignificant TE–TE correlation (> 5% FDR) but significant TE-splicing interaction effects (≤ 1%) FDR in normal lung tissues from GTEx, 3*.*06% of them had significant TE–TE correlation (5% FDR) in tumor tissues of CPTAC, higher than randomly selected gene pairs (1*.*49%). In the tumor-adjacent normal tissues of CPTAC, 4*.*30% of the gene pairs with significant TE-splicing interaction effects only from GTEx were also showing evidences of TE–TE correlations (5% FDR), again much higher than randomly selected pairs (1*.*69%). Those results showed that gene pairs with splicing-specific TE–TE correlation in normal lung tissues (i.e., TE–TE correlations in subsets of samples) from GTEx were more likely to be correlated in total expression in both tumor and adjacent normal lung tissues from CPTAC data, compared to randomly selected gene pairs. Moreover, those genes showed differential TE–TE correlations in tumor versus tumor-adjacent normal tissues. As shown in (Additional file 1: Fig. S4), those genes have greater differences in TE–TE correlations between tumor and normal tissues in CPTAC, compared to effect size differences from randomly selected gene pairs. Our results suggested that accounting for splicing-induced inter-sample variation in TE–TE correlations would not only identify co-expressed gene pairs that can be replicated in different studies and cellular-conditions; moreover, those gene pairs may have differential correlation effects in tumor-versus-normal tissues.

### Tissue-sharing patterns of TE-splicing interaction effects across 13 GTEx brain tissues

To characterize the effect-sharing patterns across multiple tissues for TE-splicing interaction effects, we studied the Alzheimer’s disease (AD) pathway from KEGG [21] and examined the patterns across 13 GTEx brain tissues (*N* = 317). There are 90 genes with at least 2 isoforms in the KEGG AD pathway. We obtained the *P*-values for the 90 × 89 gene pairs based on results from the three tests: 1) the interaction test for TE-splicing interaction effect only, 2) the joint association test for both TE–TE correlation and TE-splicing interaction, and 3) the standard correlation test, all adjusting for covariates. We analyzed data from each of the 13 GTEx brain tissues. For each tissue, we applied a 5% stratified FDR threshold to the *P*-values and obtained the significant gene pairs. We counted each gene’s outdegree in each tissue and detected the genes with 5 or more outdegree as hub genes.

**Fig. 4:**
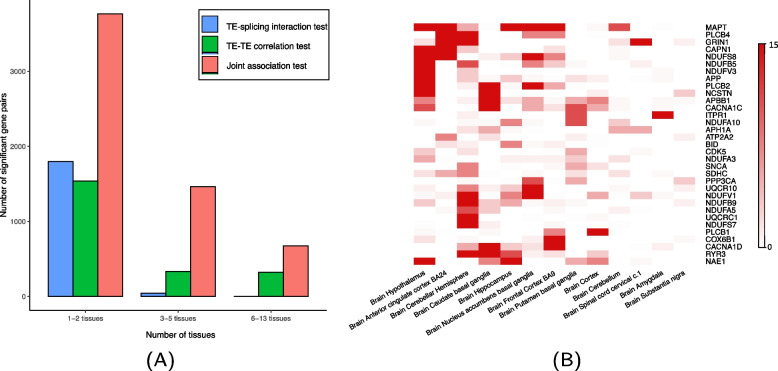
**A** Tissue-sharing patterns of significant TE-splicing interaction, TE–TE correlation, and joint association effects at the 5% stratified FDR level in 13 GTEx brain tissues for the KEGG AD pathway. **B** Tissue-sharing patterns of hub genes from AD pathway in 13 GTEx brain tissues, based on the TE-splicing interaction test. Each column is a brain tissue type. Each row is one hub gene from KEGG AD pathway. Color indicates the number of genes regulated by each hub gene with significant TE-splicing interaction effects at the 5% stratified FDR level. Only genes with significant TE-splicing interaction effects on at least 5 other genes in at least one tissue type are plotted. The gene *MAPT* (top row) has significant TE-splicing interaction effects on more than 10 other genes in multiple tissues. Other hub genes such as *PLCB2*, *NDUFS8* and *GRIN1* also have effects in 2–5 brain tissues. No cross-tissue hub genes were identified

Figure 4A showed the patterns of tissue-sharing for TE-splicing interaction effects, TE–TE correlation effects and results based on the joint association test. We found that most of the TE-splicing interaction effects are tissue-specific, with effects in only 1–2 tissues. In comparison, gene–gene TE–TE correlation effects tend to have more tissue-shared effects. The tissue-specificity of TE-splicing interaction effects echoed our previous observations that TE-splicing interactions and splicing-specific effects are less stable comparing with TE–TE correlations. Despite of the tissue-specificity of TE-splicing interaction effects, the joint test accounting for splicing-induced variation is more powerful and identified more gene pairs with effects shared across brain tissues.

In Fig. 4B, we further plotted the patterns of hub genes with TE-splicing interaction effects on multiple other genes in each brain tissue based on the interaction test at the 5% stratified FDR level. We found the gene *MAPT* to be a hub gene with > 10 regulated genes in multiple tissues. This hub gene was uniquely identified by the interaction test and it is a known gene associated with several neurodegenerative disorders including AD and Parkinson [38]. Other hub genes such as *PLCB2*, *NDUFS8* and *GRIN1* also have effects in 1–5 brain tissues. No cross-tissue hub genes were identified.

### Accounting for splicing-induced sample variation in bulk tissue data reduces potential confounding effects

In analyzing total expression data from bulk tissue, cell-type heterogeneity is a major factor contributing to inter-sample variation. Properly estimating and accounting for cellular heterogeneity are critical in reducing potential confounding and yielding valid inferences. In this work, we show that isoform composition also contributes to inter-sample variation in TE–TE correlations; and properly accounting for isoform composition could improve effect estimation and inference, and could reduce potential confounding effects in estimating gene–gene correlations from bulk tissue expression data.

We first analyzed the 38*,*146 gene pairs with significant splicing-induced inter-sample variation in TE–TE correlation in GTEx lung tissue, and compared the effect size estimates based on the analysis adjusting for all 68 covariates (serving as the silver standard here) versus those based on the analysis adjusting for 65 covariates leaving out three PEER factors [27] highly correlated with seven major cell types, including adipocytes, epithelial cells, hepatocytes, keratinocytes, myocytes, neurons, and neutrophils. The correlation of effect sizes with and without adjusting for cell type confounders (here 3 PEER factors) was 0*.*0768, with a 95% CI of (0*.*0668, 0*.*0868). Not surprisingly, when unmeasured confounding effects are inadequately adjusted, the estimated effect sizes were poorly correlated with the effect sizes estimated with the adjustment (silver standard). In contrast, after accounting for splicing-induced inter-sample variation, the correlation of effect sizes with and without adjusting for surrogate confounders (three PEERs) increased to 0*.*4361, with a 95% CI of (0*.*4280, 0*.*4442). Although isoform composition variation often does not fully capture the variation in cell-type composition, they both contribute to inter-sample variation. Properly accounting for splicing-induced variation substantially reduces inter-sample heterogeneity and improves the estimated effect sizes.

Not all the co-expressed genes were affected by the seven cell types. We further restricted the analysis to 200 gene pairs among the 38*,*146 co-expressed gene pairs whose splicing variation in the predictor gene had highest mean correlation with the seven cell types (with cell types having non-zero confounding effects). When comparing the effect sizes with and without adjusting for 3 PEER factors, the correlation was − 0*.*0461, with a 95% CI of (− 0*.*1837, 0*.*0932). When confounders (cell types) were not properly accounted for (without adjusting for the three PEERs for major cell types), effect size estimates could be biased, resulting in misleading inferences. In contrast, after adjusting for splicing variation of the predictor gene, the correlation increased to 0*.*6605, with a 95% CI of (0*.*5744, 0*.*7322). In analyzing total expression data from bulk tissues, cell type and other unmeasured confounders could lead to inter-sample heterogeneity. Splicing variation also contributes to inter-sample variation. Accounting for splicing variation may partially alleviate the potential unmeasured and inadequately adjusted confounding effects in analyzing bulk tissue expression data.

## Discussion

The majority of genes in the genome are alternatively spliced during transcription, resulting in multiple isoforms for each gene. Different isoforms of a gene may have different co-expressed genes and different regulatory effects on downstream genes. Each bulk tissue sample has a varying isoform composition for each gene, and the measured total expression levels of a gene is a mixture of isoforms-specific expression levels. In this work, we analyzed the total expression levels from bulk tissue samples to study gene–gene correlation and co-expression patterns, while accounting for splicing and isoform composition variation of each sample. We showed that splicing variation leads to substantial inter-sample variability in gene–gene correlations for many co-expressed gene pairs. Since gene–gene correlations serve as the backbone for constructing gene regulatory networks, it is essential to consider splicing variation when analyzing total expression levels of co-expressed genes. By accounting for splicing variation, our analysis results showed improved power and reproducibility for detecting and replicating co-expressed gene pairs. We also showed that adjusting for splicing variation may reduce potential confounding effects in studying total expression levels from bulk tissue data.

To model the inter-sample variation in isoform composition and their interactions with TE levels, we adopted a linear mixed-effects model with the TE of the putative regulated/co-expressed gene as the response, and the TE of the putative regulator gene as predictor, allowing the TE–TE effects to vary across samples with inter-sample variation proportional to covariance of isoform composition. Instead of quantifying isoform-level expression, we used Leafcutter and estimated intron excision ratios as surrogate variables for relative isoform proportions. We further used a variance-component score test for testing splicing-induced inter-sample variation in TE–TE correlations. The test can be reduced to an omnibus interaction test for interaction effects of IER and TE of a gene on the response gene when there are only two or a few isoforms; and when there are many isoforms, the variance component is well-powered to detect splicing-induced inter-sample variation in TE–TE effects. Moreover, by jointly testing for fixed (TE–TE correlation) and random-effects (splicing-induced inter-sample variation in TE–TE correlation) weighted by the estimated variance components, we showed that power was improved in detecting co-expressed gene pairs. The variance-component score tests for random slope and the joint association test for fixed and random slope have been used in other applications, for example in detecting gene-environment interactions involving multiple environments [20, 30]. Similar models with random-intercepts have also been used in adjusting for population substructure or encrypted genetic relatedness [39–41] in genetic association studies. Here we used it to account for splicing-induced inter-sample variation. In the precision medicine era, the model and methods can be broadly used to test for and adjust for inter-sample variation and heterogeneity in effects due to a suspected large set of covariates.

There are some caveats in our analyses. In this work, we analyzed only ∼ 3000 genes and over 10 M pairs of genes in the genome. However, we believe that the patterns observed and conclusions from our analysis could be extended to all genes in a genome-wide analysis. Additionally, in reconstructing the TE–TE network, we used a simple stratified FDR approach and correlation in residuals among all genes in the network was ignored. Other widely used methods such as graphical lasso methods could have been used. We chose a simple network reconstruction method because we observed that the residual correlations among genes within the pathway were substantially reduced after accounting for splicing-induced inter-sample variation. In other words, after accounting for splicing, there are much weaker multivariate gene–gene correlations, and a multivariate network methods may not be needed. Further research on a joint network accounting for splicing-induced inter-sample variation in each node will be explored in future research.

## Conclusions

In this work, by analyzing the total expression levels for pairs of genes from GTEx (V8) lung and brain tissue data and accounting for inter-sample splicing variation, we showed that there are prevalent isoform-specific regulatory effects in the genome. Moreover, variation in isoform composition among different samples lead to substantial inter-sample variation in TE–TE correlations for co-expressed gene pairs. By analyzing the TE–TE correlations of genes in an independent dataset from lung adenocarcinoma cancer patients from CPTAC, we found that gene pairs with significant isoform-specific TE–TE correlations in GTEx had higher-than-random proportions of TE–TE correlations in tumor and tumor-adjacent normal tissues, with differential TE–TE correlation effect sizes. Further studying effects-sharing patterns of an AD pathway across 13 GTEx brain tissues, we found isoform-specific TE–TE correlations (TE-splicing interactions) tend to be tissue-specific and less stable than TE–TE correlations, even in functionally related brain tissues. Despite of the tissue-specificity of splicing-induced variation, properly accounting for splicing-induced inter-sample variation in a joint association test would improve the power to detect co-expressed gene pairs across studies and cellular conditions. Furthermore, with adjustment of splicing variation, the estimated TE–TE correlation and inference are more robust and less prone to confounding effects from cell type and other unknown confounders. Moreover, by examining co-expressed gene pairs within specific KEGG pathways, we characterized the patterns of the hub genes with splicing-induced inter-sample variation affecting multiple other genes. We identified *TP53*, a known tumor suppressor gene, as the biggest TE-splicing interaction hub in the network based on the interaction tests within the small cell lung cancer pathway, using data from normal lung tissues of GTEx. The gene was not a major hub in the networks constructed based on the standard correlation test nor the joint association test. Our results also showed that splicing hub genes with isoform-specific effects on many other genes tend to have smaller communities.

## Methods

We used a linear mixed-effects model to capture both the fixed effects from the TE of a putative regulator gene on the TE of a response gene, as well as the random effects across samples in TE–TE correlations due to sample-specific splicing variation.

Let ${\varvec{y}}$ and ${\varvec{x}}$ be the vectors of mean-centered gene expression levels over $n$ samples for the response gene and the putative regulator gene, respectively, or for two co-expressed genes. Note that applying the proposed test to genes without meancentering is still valid and can control the type I error rates, but may impose different weights on the interaction variation for genes with different means. Thus we recommend mean-centering the expression levels. Let $\mathbf{Z}$ denote the fixed-effect design matrix of $p$ covariates including an intercept, and $\text{diag}(\cdot )$ is a function that transforms a vector to a diagonal matrix. Consider the linear mixed-effects model:

1 $${\varvec{y}}={\varvec{x}}{\beta }_{1}+\text{diag}({\varvec{x}}){{\varvec{\beta}}}_{2}+\mathbf{Z}{\varvec{\gamma}}+{\varvec{u}}+{\varvec{\varepsilon}},$$

where ${\beta }_{1}$ captures the fixed effects between the TE levels of the two genes across all samples, ${{\varvec{\beta}}}_{2}={\left({\beta }_{2}^{1},\cdots ,{\beta }_{2}^{n}\right)}^{\top}$ represents the random slope, i.e., inter-sample variation in TE–TE correlation, and ${\varvec{\gamma}}$ is the effect of covariates. We assume that ${{\varvec{\beta}}}_{2}$ follows a multivariate normal distribution, $N\left(0,{\sigma }_{\text{Int }}^{2}{\varvec{\Sigma}}\right)$, where ${\varvec{\Sigma}}$ is the covariance matrix of multiple IERs (surrogate for isoforms) of the putative regulator gene $X$. In other words, we assume that the variance of random slope is proportional to the covariance matrix of isoform composition of gene $X$. Samples with similar isoform compositions of gene $X$ would have similar TE–TE correlations. The random intercept ${\varvec{u}}\sim N\left(0,{\sigma }_{u}^{2}{\varvec{\Sigma}}\right)$ together with the fixed effect intercept captured the splicing effects of gene $X$ on the TE levels of gene $Y$, and those effects could be due to post-translational regulation and is not the main interest of the current work. And ${\varvec{\varepsilon}}\sim N\left(0,{\sigma }_{e}^{2}\mathbf{I}\right)$ is the error term.

A variance-component-based interaction test for testing splicing induced inter-sample variation in TE–TE correlation.

Similar to SKAT [30] and many other tests for interactions [20], here we adopted a variance-component score test to test whether there was inter-sample variation in TE–TE correlation induced by splicing variation in gene *X*. Specifically, in Eq. 1, we test ${H}_{0}:{\sigma }_{\text{Int }}^{2}=0$. The variance-component score test is well-powered when there are many variables contributing to the variation in random slope [42]. Here we used it to test for the variance component of random slope induced by sample-level variation in isoform composition of a gene. The test statistics is given by

2 $$Q = {\left({\varvec{y}} - \widehat{{\varvec{y}}}\right)}^{\text{T}}{{\varvec{V}}}^{-1} \text{diag} \left({\varvec{x}}\right) \sum \text{diag} \left({\varvec{x}}\right) {{\varvec{V}}}^{-1} \left({\varvec{y}} - \widehat{{\varvec{y}}}\right),$$

where $\widehat{{\varvec{y}}}$ is the fitted value of ${\varvec{y}}$ under null hypothesis of no random slope with ${\sigma }_{\text{Int }}^{2}=0$. Under null hypothesis, ${\widehat{\sigma }}_{u}$ and ${\widehat{\sigma }}_{e}$ are estimated using MLE [41, 43], and $\mathbf{V} = {\widehat{\sigma }}_{u}^{2}{\varvec{\Sigma}}+{\widehat{{\varvec{\sigma}}}}_{{\varvec{e}}}^{2}\mathbf{I}$ is the estimated total covariance. Since ${\varvec{\Sigma}}$ is a real symmetric matrix, it could be decomposed into a canonical form based on spectral theorem. Therefore, the test statistic $Q$ follows a weighted sum of chi-squared distributions under null hypothesis [30, 44, 45]. Specifically, the weights are the eigenvalues of ${\varvec{\Sigma}}$. The $P$-value was computed using Liu's method [46]. The method is known to be robust to violations of normal assumptions [20, 30]. The matrix ${\varvec{\Sigma}}$ models the sample covariance matrix based on isoform-composition. Generally, if there is a set of covariates that may contribute to inter-sample variation in effects, one may estimate the sample-sample covariance based on the set of covariates as ${\varvec{\Sigma}}$, and apply the above variance-component score test. And the number of covariates can be much larger than the sample size. Similar and related tests have been applied to detect gene-environment interactions involving a wide range of environmental variables [20, 30], and to adjust for encrypted genetic relatedness [39–41] in genetic association studies.

A joint association test for TE–TE correlation and inter-sample variation in TE–TE correlation.

To jointly test for fixed and random slopes in the above mixed-effects model, we applied the joint association test proposed in StructLMM [20]. We rewrote the model as

3 $$\begin{array}{cc}& y={\varvec{x}}{\beta }_{1}+\text{diag}({\varvec{x}}){{\varvec{\beta}}}_{2}+{\varvec{Z}}{\varvec{\gamma}}+{\varvec{u}}+{\varvec{\varepsilon}},\\ & =\text{diag}({\varvec{x}})\left({\beta }_{1}1+{{\varvec{\beta}}}_{2}\right)+{\varvec{Z}}{\varvec{\gamma}}+{\varvec{u}}+{\varvec{\varepsilon}}.\end{array}$$

The variance of ${\beta }_{1}1+{{\varvec{\beta}}}_{2}$ is

4 $$\text{Var}\left({\beta }_{1}1+{{\varvec{\beta}}}_{2}\right)={\sigma}_{X}^{2}1{1}^{\top}+{\sigma }_{\text{Int }}^{2}{\varvec{\Sigma}}={\sigma }^{2}\left((1-\rho )11^{\top}+\rho{\varvec{\Sigma}}\right),$$

where ${\sigma }^{2}$ represents the total variance of the effect of gene $X$ on $Y$ from both fixed and random slope. And

5 $$=\frac{{\sigma }_{{\text{Int}} \, }^{2}}{{\sigma }_{{\text{Int}} \, }^{2}+{\sigma }_{X}^{2}}$$

is the proportion of splicing-induced inter-sample variation among the total contribution of variation in *X* to the total variation in *Y*, i.e., the relative contribution of random effects in the total variation in *X* when explaining the variation in *Y*. By estimating *ρ*, we can test for the joint effect from *X* on *Y* while also specifying and weighing by the relative contributions by fixed effects of *X* and by splicing-induced random-effects. We adopted a quadratic test statistic,

6 $$\begin{array}{cc}& Q=({\varvec{y}}-\widehat{{\varvec{y}}}{)}^{\top }{{\varvec{V}}}^{-1}\text{diag}({\varvec{x}})\cdot \left((1-\rho )1{1}^{\top }+\rho{\varvec{\Sigma}}\right)\cdot \\ & \text{diag}({\varvec{x}}){{\varvec{V}}}^{-1}({\varvec{y}}- \widehat{{\varvec{y}}}).\end{array}$$

Similar to the test statistic for interaction test, here $Q$ also follows a mixture of ${\chi }^{2}$ distributions. We calculated the corresponding *P*-values using methods mentioned above. The association test generalizes previous methods [47, 48] where a two degree of freedom test was performed. When the dimension of isoforms of a gene is large with unknown relative contribution of fixed and random effects, the joint association test is well-powered and efficient.

## Supplementary Information

Additional file 1: Fig S1. The histogram of *P*-values obtained from the joint association test for fixed and random effects, i.e., a joint association test for TE-TE and TE-isoform interaction, accounting for splicing-induced sample-level variation in the TE-TE effects. Fig S2. Network constructed based on the joint association tests and standard correlation tests within the small cell lung cancer pathway from KEGG. (A) The network based on the standard TE-TE correlations adjusting for covariates showed six major hub genes, where the biggest ones were *BCL2L1 *and *LAMA2*. (B) The network based on the joint association test showed more dense structure with more edges and hub genes. The gene that had the maximal outdegree was *LAMB2*, with effects on 45 other genes, and was a shared hub with the network based on the correlation tests and interaction tests. Fig S3. An examination of the degree centrality distribution based on the interaction, the correlation, and the joint association tests across all 158 KEGG pathways. An outdegree of a gene was defined as how many other genes were significant using this gene as putative regulator gene. For correlation tests, outdegree was the same as indegree. (A) The average outdegrees of genes increased linearly with pathway size. The TE-splicing interaction networks had generally lower outdegrees than the networks based on the correlation and the joint association tests. Networks based on correlation tests had higher mean outdegrees than TE-splicing interaction networks in 113 pathways while networks based on joint association tests had higher mean outdegree than TE-splicing interaction networks in 151 pathways. (B) Comparison of pathway-size-adjusted mean outdegrees across 158 KEGG pathways. The mean of outdegree of each pathway was calculated by dividing the sum of all outdegrees in the pathway by the number of genes in this pathway. Bars in red represents the mean of outdegrees per gene based on the joint association test. Orange and yellow corresponded to mean outdegree per gene based on the correlation and the interaction test, respectively. The bars were sorted based on the mean outdegrees of the joint association tests. The bar plot in the left shows the top 79 pathways that had highest mean outdegrees in the joint association tests. The bar plot in the right side shows the size-adjusted outdegree distributions of the rest of the 79 pathways. Fig S4. The Quantile-Quantile (QQ) plot of −log_10_(*P*-value) for testing differential co-expression between tumor versus tumor-adjacent normal tissues for gene pairs identified from GTEx based on the interaction test versus randomly-selected genes. For gene X and gene Y with significant TE-splicing interaction of X on Y at the 5% FDR in GTEx, we calculated the *P*-value for testing differential correlation between TE_X_ on TE_Y_ after adjusting for covariates in tumor samples versus tumor-adjacent normal samples of CPTAC. Compared to randomly selected gene pairs, pairs detected by interaction tests in GTEx showed greater difference between tumor tissues and tumor-adjacent normal tissues.

## Data Availability

GTEx (V8) expression data can be downloaded through (https://www.gtexportal.org). CPTAC expression data can be downloaded through dbGap (study id phs001287.v3.p3). Analysis code has been released through https://github.com/ylustat/AlternativeSplicing.

## Abbreviations

TE: Total expression
GTEx: The Genotype-Tissue Expression project
AS: Alternative splicing
CPTAC: Clinical Proteomic Tumor Analysis Consortium
IER: Intron excision ratio
IR: Isoform ratio
