# Supplementary material for: Alternative splicing induces sample-level variation in gene–gene correlations
Source: BMC Genomics. 2024 Dec 11;23(Suppl 4):867. doi: 10.1186/s12864-024-11118-z (PMC11633002; doi:10.1186/s12864-024-11118-z)
Supplement: Supplementary file 1 — Additional file 1: Fig S1. The histogram of P-values obtained from the joint association test for fixed and random effects, i.e., a joint association test for TE-TE and TE-isoform interaction, accounting for splicing-induced sample-level variation in the TE-TE effects. Fig S2. Network constructed based on the joint association tests and standard correlation tests within the small cell lung cancer pathway from KEGG. (A) The network based on the standard TE-TE correlations adjusting for covariates showed six major hub genes, where the biggest ones were BCL2L1 and LAMA2. (B) The network based on the joint association test showed more dense structure with more edges and hub genes. The gene that had the maximal outdegree was LAMB2, with effects on 45 other genes, and was a shared hub with the network based on the correlation tests and interaction tests. Fig S3. An examination of the degree centrality distribution based on the interaction, the correlation, and the joint association tests across all 158 KEGG pathways. An outdegree of a gene was defined as how many other genes were significant using this gene as putative regulator gene. For correlation tests, outdegree was the same as indegree. (A) The average outdegrees of genes increased linearly with pathway size. The TE-splicing interaction networks had generally lower outdegrees than the networks based on the correlation and the joint association tests. Networks based on correlation tests had higher mean outdegrees than TE-splicing interaction networks in 113 pathways while networks based on joint association tests had higher mean outdegree than TE-splicing interaction networks in 151 pathways. (B) Comparison of pathway-size-adjusted mean outdegrees across 158 KEGG pathways. The mean of outdegree of each pathway was calculated by dividing the sum of all outdegrees in the pathway by the number of genes in this pathway. Bars in red represents the mean of outdegrees per gene based on the joint association test. Orange and [file 12864_2024_11118_MOESM1_ESM.pdf]

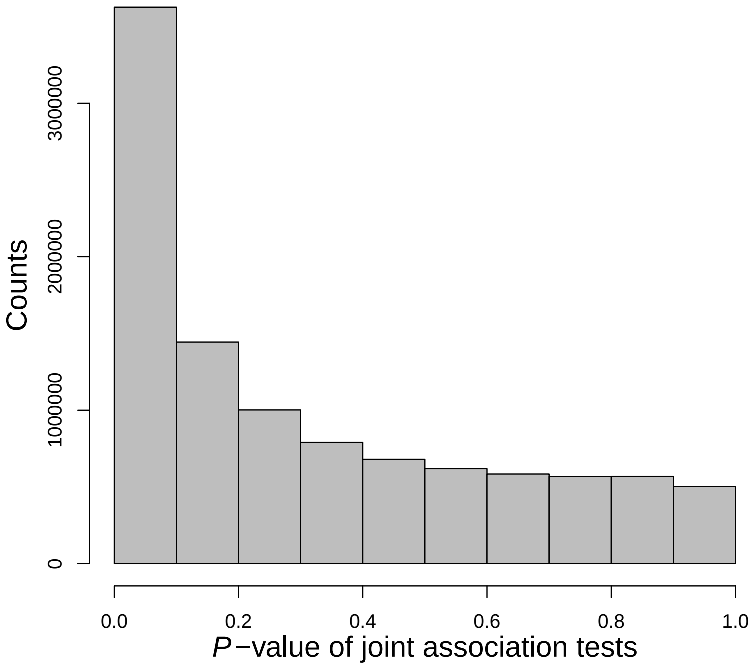

Figure S1: The histogram of P-values obtained from the joint association test for fixed and random effects, i.e., a joint association test for TE-TE and TE-isoform interaction, accounting for splicing-induced sample-level variation in the TE-TE effects.

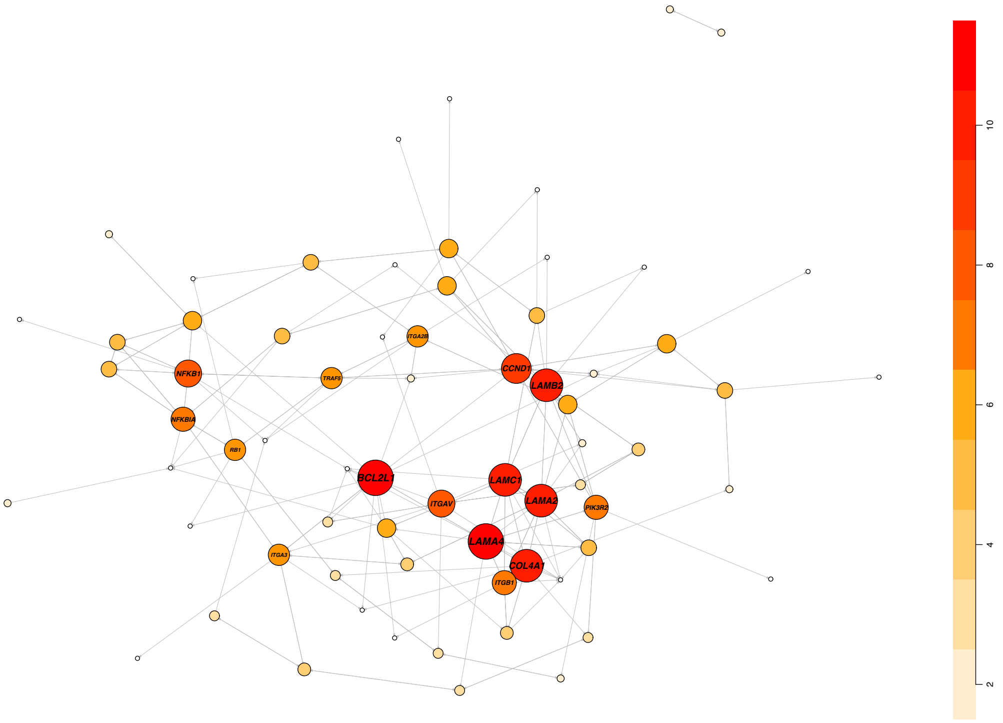

(A)

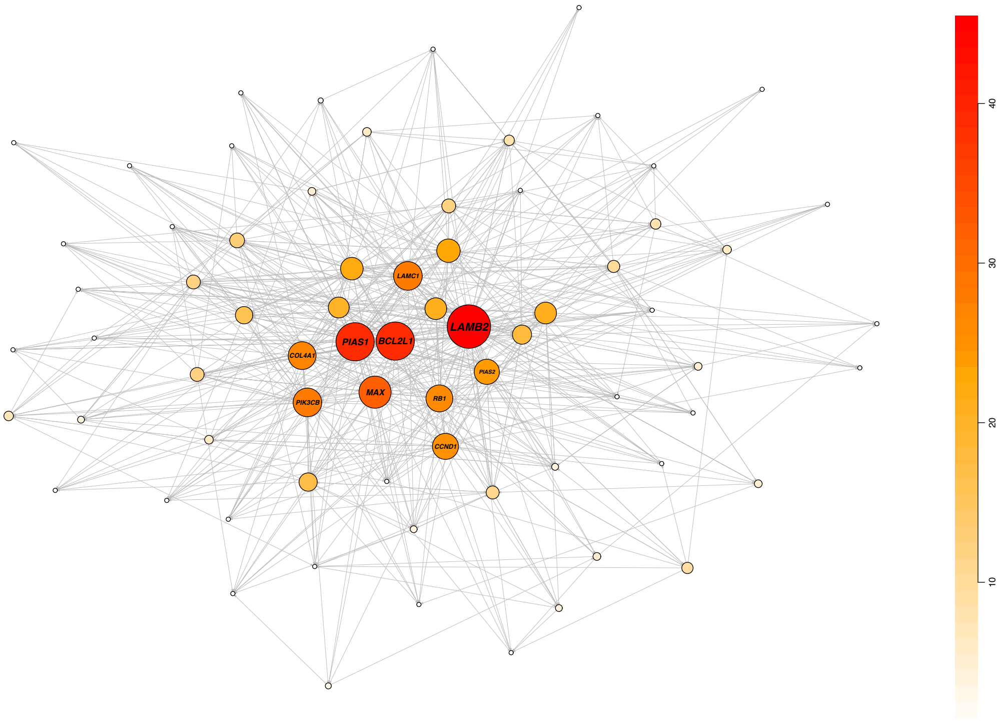

(B)

Figure S2: Network constructed based on the joint association tests and standard correlation tests within the small cell lung cancer pathway from KEGG. (A) The network based on the standard TE-TE correlations adjusting for covariates showed six major hub genes, where the biggest ones were *BCL2L1* and *LAMA2*. (B) The network based on the joint association test showed more dense structure with more edges and hub genes. The gene that had the maximal outdegree was *LAMB2*, with effects on 45 other genes, and was a shared hub with the network based on the correlation tests and interaction tests.

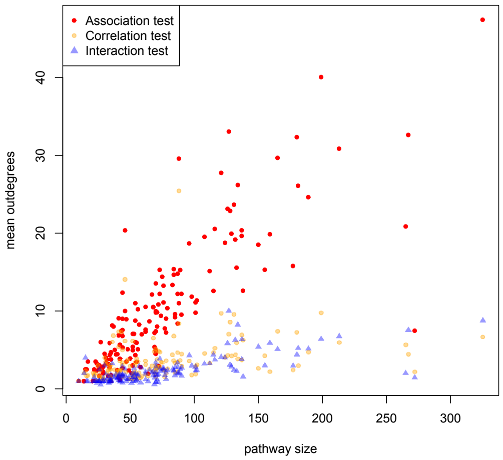

(A)

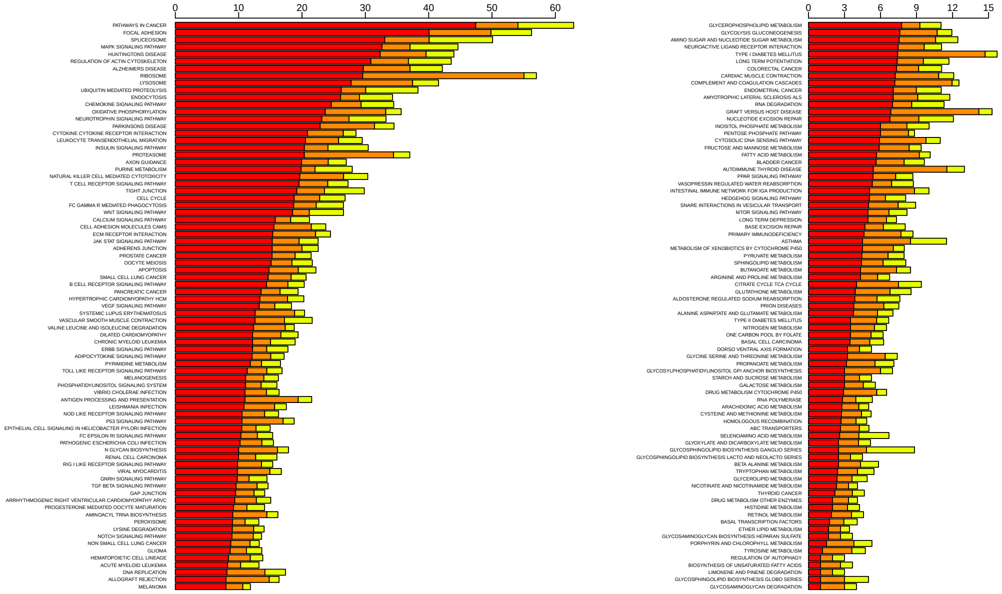

(B)

Figure S3: An examination of the degree centrality distribution based on the interaction, the correlation, and the joint association tests across all 158 KEGG pathways. An outdegree of a gene was defined as how many other genes were significant using this gene as putative regulator gene. For correlation tests, outdegree was the same as indegree. (A) The average outdegrees of genes increased linearly with pathway size. The TE-splicing interaction networks had generally lower outdegrees than the networks based on the correlation and the joint association tests. Networks based on correlation tests had higher mean outdegrees than TE-splicing interaction networks in 113 pathways while networks based on joint association tests had higher mean outdegree than TE-splicing interaction networks in 151 pathways. (B) Comparison of pathway-size-adjusted mean outdegrees across 158 KEGG pathways. The mean of outdegree of each pathway was calculated by dividing the sum of all outdegrees in the pathway by the number of genes in this pathway. Bars in red represents the mean of outdegrees per gene based on the joint association test. Orange and yellow corresponded to mean outdegree per gene based on the correlation and the interaction test, respectively. The bars were sorted based on the mean outdegrees of the joint association tests. The bar plot in the left shows the top 79 pathways that had highest mean outdegrees in the joint association tests. The bar plot in the right side shows the size-adjusted outdegree distributions of the rest of the 79 pathways.

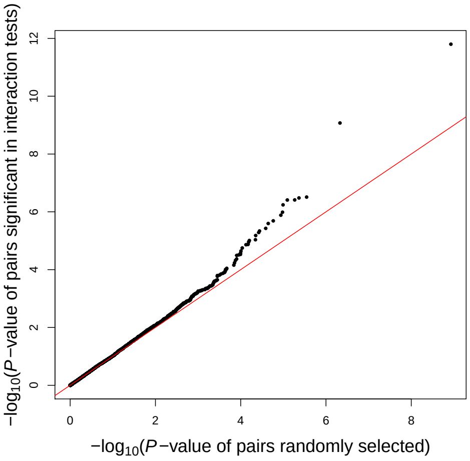

Figure S4: The Quantile-Quantile (QQ) plot of $-\log_{10}(P\text{-value})$ for testing differential co-expression between tumor versus tumor-adjacent normal tissues for gene pairs identified from GTEx based on the interestion test versus randomly-selected genes. For gene X and gene Y with significant TE-splicing interaction of X on Y at the 5% FDR in GTEx, we calculated the P-value for testing differential correlation between TE_X on TE_Y after adjusting for covariates in tumor samples versus tumor-adjacent normal samples of CPTAC. Compared to randomly selected gene pairs, pairs detected by interaction tests in GTEx showed greater difference between tumor tissues and tumor-adjacent normal tissues.
